# Active Preconditioning With Blood Flow Restriction or/and Systemic Hypoxic Exposure Does Not Improve Repeated Sprint Cycling Performance

**DOI:** 10.3389/fphys.2019.01393

**Published:** 2019-11-14

**Authors:** Mathias R. Aebi, Sarah J. Willis, Olivier Girard, Fabio Borrani, Grégoire P. Millet

**Affiliations:** ^1^ISSUL, Institute of Sport Sciences, Faculty of Biology and Medicine, University of Lausanne, Lausanne, Switzerland; ^2^Aeromedical Center (AeMC), Swiss Air Force, Dübendorf, Switzerland; ^3^Murdoch Applied Sports Science (MASS) Laboratory, Murdoch University, Perth, WA, Australia

**Keywords:** altitude, BFR, blood volume, near-infrared spectrometry, oxygenation, RSE

## Abstract

**Purpose:**

The aim of this study was to evaluate the effects of active preconditioning techniques using blood flow restriction or/and systemic hypoxic exposure on repeated sprint cycling performance and oxygenation responses.

**Methods:**

Participants were 17 men; 8 were cycle trained (T: 21 ± 6 h/week) and 9 were untrained but physically active (UT). Each participant completed 4 cycles of 5 min stages of cycling at 1.5 W⋅kg^–1^ in four conditions [Control; IPC (ischemic preconditioning) with partial blood flow restriction (60% of relative total occlusion pressure); HPC (hypoxic preconditioning) in normobaric systemic hypoxia (F_I_O_2_ 13.6%); and HIPC (hypoxic and ischemic preconditioning combined)]. Following a 40 min rest period, a repeated sprint exercise (RSE: 8 × 10 s sprints; 20 s of recovery) was performed. Near-infrared spectroscopy parameters [for each sprint, change in deoxyhemoglobin (Δ[HHb]), total hemoglobin (Δ[tHb]), and tissue saturation index (ΔTSI%)] were measured.

**Results:**

Trained participants achieved higher power outputs (+10–16%) than UT in all conditions, yet RSE performance did not differ between active preconditioning techniques in the two groups. All conditions induced similar sprint decrement scores during RSE in both T and UT (16 ± 2 vs. 23 ± 9% in CON; 17 ± 3 vs. 19 ± 6% in IPC; 18 ± 5 vs. 20 ± 10% in HPC; and 17 ± 3 vs. 21 ± 5% in HIPC, for T and UT, respectively). During the sprints, Δ[HHb] was larger after IPC than both HPC and CON in T (*p* < 0.001). The Δ[tHb] was greater after HPC than all other conditions in T, whereas IPC, HPC, and HIPC induced higher Δ[tHb] than CON in UT.

**Conclusion:**

None of the active preconditioning methods had an ergogenic effect on repeated sprint cycling performance, despite some specific hemodynamic responses (e.g., greater oxygen extraction and changes in blood volume), which were emphasized in the trained cyclists.

## Introduction

There is renewed interest for the so-called “ischemic preconditioning” (IPC) strategy and its application in the athletic field for performance improvement (Incognito et al., 2015). IPC involves intermittent circulatory cycles of vascular occlusion and reperfusion of blood flow (local hypoxia) to prepare and protect the body’s cells against successive events of similar or greater ischemic/hypoxic stress (Das and Das, 2008). Previous researchers have typically implemented IPC at rest in athletes via total occlusion (220 mm Hg) with 3 × 5 min occlusion-reperfusion cycles (Groot et al., 2009; Clevidence et al., 2012).

Repeated sprinting is an exercise model that includes the repetition of short “all-out” efforts (<10 s) with incomplete recoveries (<60 s or work to rest ratio <1:4) (Girard et al., 2012). In the literature, contradictory results exist regarding the effects of IPC on repeated sprint exercise (RSE) performance. Repeated sprint cycling performance has been shown to improve by 1–2% when IPC is performed at rest for 30–45 min preceding RSE (Patterson et al., 2014; Lalonde and Curnier, 2015). For instance, peak and mean power outputs were improved during the first three 6 s cycling sprints in a series of twelve (Patterson et al., 2014). In contrast, IPC induced similar performance outcomes as a sham during either 5 × 6 s repeated sprints (Gibson et al., 2015) or 6 × 6 s sprints and a 30 s Wingate test (Lalonde and Curnier, 2015).

Some of the variation in the effects of IPC on RSE may be attributed to the differences in the studied populations. No research has systematically examined to compare individuals of various training status in terms of performance and responses to exercises following IPC (Incognito et al., 2015; Salvador et al., 2016). Participant populations of previous studies have varied considerably and included recreationally active, trained, or elite athletes (Groot et al., 2009; Jean-St-Michel et al., 2011; Gibson et al., 2015; Lalonde and Curnier, 2015). Thus, the variability of exercise performance in response to IPC is still poorly understood. In a recent review, it is suggested that there are responders and non-responders (Incognito et al., 2015), however, the interpretation of mean group data is confounded by small sample sizes and the participant heterogeneity between studies. Moreover, there is a lack of comparison between diverse populations in the literature. Recently, differences in muscle oxygenation responses, as measured non-invasively by near infrared spectroscopy (NIRS), were found between trained (T) and untrained (UT) individuals (Soares et al., 2018). Specifically, the re-oxygenation slope on the tibialis anterior muscle of the leg was steeper during endurance exercise in trained vs. untrained following 5 min of vascular occlusion, suggesting positive vascular responses in endurance trained individuals (Soares et al., 2018). One may hypothesize that there are putative differences (1, fiber type distribution/recruitment; 2, cardiac output and leg blood flow; and 3, hypoxemia) that justify investigating the respective influence of IPC or hypoxic preconditioning (HPC) between trained and untrained subjects. In particular, (1) one mechanism may come from the differences in muscle fiber recruitment, where fast twitch (FT) fibers are known to likely benefit from increased perfusion (Faiss et al., 2013) induced by systemic hypoxia; (2) trained subjects have higher V̇O_2__max_, cardiac output, and leg blood flow than their lower level counterparts (Montero and Díaz-Cañestro, 2015) along with greater consequential shear stress and/or NO-mediated vasodilatory (post-HPC with systemic hypoxia) or hyperemic (post-IPC with BFR) responses; and (3) the prevalence and magnitude of exercise-induced full level of hypoxemia (i.e., decrease in oxygen saturation and arterial oxygen content), which is larger in endurance-trained athletes than in untrained participants (Mollard et al., 2007). Hypoxemia may influence the hemodynamic responses post-HPC between trained and untrained participants. Overall, one may therefore speculate that trained participants would benefit to a larger extent than untrained from HPC, while IPC would induce similar responses between these two groups.

Rather than using “traditional” IPC (i.e., total occlusion) to induce full level of ischemia at rest, the present study implemented an active preconditioning using partial blood flow restriction (BFR), in order to limit the blood flow to the working muscles (Loenneke et al., 2010). This partial occlusion is needed for the current form of IPC (i.e., active preconditioning implying cycling exercise). To our knowledge, previous studies investigating the effects of IPC on exercise performance have performed complete occlusion (above systolic blood pressure) in order to occlude the blood flow in the limb (Groot et al., 2009; Lalonde and Curnier, 2015; Thompson et al., 2018). This practice increases the adenosine level and ATP-sensitive potassium channels enhancing vasodilation. Moreover, it was hypothesized that IPC may improve the tolerance to hypoxia during high-intensity exercise, and therefore improve short duration exercise performance (Crisafulli et al., 2011). These mechanisms could thus be beneficial for high-intensity exercise performance. The present study investigated whether an active preconditioning using BFR altering oxygenation could improve repeated sprint cycling performance and associated hemodynamic responses.

Hypoxic preconditioning consists of acute exposure to systemic hypoxia separated with rest periods in normoxia. Until now, HPC prior to exercise performance has been primarily used as a method for pre-acclimatization to altitude (Benoit et al., 1992). To our knowledge, HPC has never been implemented during exercise in a similar protocol as IPC before completing a performance test. With systemic hypoxia comes a reduced oxygen availability exacerbating muscle deoxygenation during exercise (Oguri et al., 2008; Yamaguchi et al., 2019). An autonomic vasodilation may cause an increase in blood flow to the muscle tissue in order to maintain oxygen delivery (Casey and Joyner, 2012; Dinenno, 2016). In contrast, blood flow restricted exercise induces both ischemia and local hypoxia and this may induce an effect on vascular/endothelial function due to vascular resistance and vessel diameter adjustment, accumulation of metabolites (nitric oxide, adenosine, prostaglandins, hydrogen ions, etc.) and/or indirect sympathetic activation. Due to cuff application in IPC conditions, the demand for increased blood flow cannot be met with similar mechanisms as during systemic hypoxia (i.e., hypoxia-induced vasodilation). Altogether, HPC and IPC are different methods (systemic hypoxia via lower F_I_O_2_ versus local hypoxia via greater vascular resistance and lower blood flow) with different intrinsic mechanisms (metabolic vasodilation to increase blood flow for oxygen delivery versus greater vascular challenge for blood flow regulation). During HPC and IPC, a hypoxic environment may exist, in which alterations of vascular conductance and blood flow are present. IPC also promotes vasodilation through the reactive hyperemic effect (Enko et al., 2011). Recent research has shown that ischemic conditions elicit greater changes in tissue perfusion (via blood volume) than a control without BFR during cycling RSE (Willis et al., 2018). However, it remains unknown if the so-called active hypoxic-ischemic preconditioning (HIPC) that consists of combining HPC and IPC would potentiate or blunt the respective effects of systemic localized hypoxia on hemodynamic responses (Willis et al., 2016).

Therefore, the purpose of the present study was to investigate separate and combined effects of partial BFR and hypoxic exposure during an active preconditioning on repeated sprint cycling exercise and muscle oxygenation in trained and untrained individuals. First, it was expected that active preconditioning with systemic hypoxia (HPC) or with BFR (IPC) would lead to better performance during RSE due to improved muscle oxygenation responses (increased oxygen utilization, increased blood volume) than a control or in combination (HIPC). Moreover, it was hypothesized that trained participants would have beneficial oxygenation responses with IPC and even more so with HPC, when compared with untrained participants.

## Materials and Methods

### Participants

Seventeen healthy active male subjects [age (mean ± SD) = 25 ± 2 years, body mass = 71.3 ± 7.7 kg, height = 180.3 ± 5.5 cm] volunteered to participate in this study. All subjects were active and answered a training history questionnaire prior to volunteering for the study. Participants were split into two groups: trained (T) and untrained (UT) based on weekly cycling hours (21.0 ± 6.1 vs. 0.6 ± 1.1 h for T and UT, respectively). T were trained cyclists, while UT were active individuals practicing other sports. Subjects were naive about the effects of BFR and hypoxia and had not experienced these conditions during exercise in the previous 3 months. Participants gave written consent after being informed about the potential risks and procedures of the protocol and the study followed the seventh Declaration of Helsinki (2013) as approved by the Ethical Commission for Human Research (CER-VD 138/15).

### Study Design

A randomized, single-blinded, repeated measures design was used for this study. Participants completed a familiarization session before four experimental trials all separated with a minimum of 3 days and maximum of 7 days and conducted at the same time of day (±2 h). Preconditioning protocol cycles were implemented actively while pedaling on an ergocycle with two bilateral-leg partial occlusion conditions (30 mm Hg and 60% of relative total occlusion pressure; T: 123.9 ± 5.6 mm Hg, UT: 111.1 ± 5.6 mm Hg) and two environmental conditions [normoxia and normobaric hypoxia (F_I_O_2_ 20.9% and 13.6%, respectively)] consisting of four randomized preconditioning conditions: Control (CON, 30 mm Hg/20.9% F_I_O_2_), partial blood flow restriction (IPC, 60% occlusion/20.9% F_I_O_2_), hypoxia (HPC, 30 mm Hg occlusion/13.6% F_I_O_2_), and partial ischemia added to hypoxia (HIPC, 60% occlusion/13.6% F_I_O_2_). Occlusion pressure was set at 60% of individual maximal occlusion pressure to induce similar muscle deoxygenation between IPC and HPC during the preconditioning phase based on several pilot testing sessions investigating cardiovascular (heart rate) and oxygenation (NIRS) responses. Moreover, preconditioning was performed actively on an ergocycle, as opposed to seated at rest in other studies (Groot et al., 2009; Clevidence et al., 2012) and with partial occlusion rather than total occlusion. The intensity was relative to body mass (1.5 W/kg) during active preconditioning phases for all participants. To minimize the placebo effect, participants were informed that two different occlusion pressures were tested and that both may have an impact on exercise performance. RSE consisted of eight, 10 s sprints with 20 s of active recovery on the bike.

### Familiarization

Participants first visited the laboratory for a familiarization visit that began with anthropometric and position measurements of the bike. Automated cuffs (11 × 85 cm cuff size, 10 × 41 cm bladder size, SC10D Rapid Version Cuff, D. E. Hokanson, Inc., Bellevue, WA, United States) were placed on the most proximal region of the lower limb for measurement of the total occlusion pressure. Pulse elimination pressure was measured in seated rest (on a chair) by gradually increasing the occlusion pressure until a point when no blood flow was observed in femoral artery using a linear Doppler ultrasound probe (L12-5L60N) with Echo Wave II 3.4.4 software (Telemed Medical Systems, Lithuania, Telemed Ltd., Milan, Italy), as measured two or three times for accuracy, with approximately 2 min between trials (Gualano et al., 2010). Finally, subjects were familiarized with the protocol and equipment and completed the RSE (described in detail below) on an electronically braked cycling ergometer (Lode Excalibur Sport Ergometer, Lode B.V., Netherlands).

### Experimental Trials

Upon arrival, participants completed a warm-up composed of 5 min at 50 W followed by 5 min at 100 W at 85 rpm. Subsequently, participants were fitted with a mask to simulate altitude (Everest Summit II Generator, Hypoxico Inc., New York, NY, United States and bilateral automated cuffs (E20/AG101 Rapid Cuff Inflation System, D. E. Hokanson, Inc., Bellevue, WA, United States) for the purpose of the active preconditioning treatment. The active preconditioning consisted of four cycles of 5 min of cycling at 1.5 W/kg (105.9 ± 7.0 vs. 108.6 ± 14.2 W for T and UT) at a cadence of 85 rpm alternated by 5 min of passive recovery (seated on the ergocycle). Four cycles of 5 min preconditioning alternated with 5 min rest was chosen in relation with previous studies (Jean-St-Michel et al., 2011; Griffin et al., 2018). However, preconditioning was performed actively on an ergocycle rather than at rest. After each of the four stages, participants indicated their overall and peripheral perceived discomfort in legs and breathing (rating of perceived exertion, RPE, 6–20).

Between the end of last preconditioning cycle and the start of the RSE, a 40 min rest period began with a 20 min passive rest with *ad libitum* water consumption. Then, 20 min before starting the RSE, subjects performed a standardized warm-up in normoxia consisting of 5 min cycling at 100 W, followed by four submaximal sprints at approximately 60, 70, 80, and 100% of perceived maximal effort with 3 min of recovery between. The purpose of this second warm-up was to avoid potential injury during RSE and to assess an isolated maximal sprint to monitor pacing during RSE (Girard et al., 2012). RSE started 5 min after this second warm-up.

During RSE, sprints were performed in normoxia without BFR on the cycling ergometer with a fixed resistance (torque factor of 0.8 Nm.kg^–1^) and in “Wingate mode.” Shoe clips were used to ensure pedal contact and strong encouragement was provided. All cycling bouts were initiated from a rolling start, with subjects seated on the bike and targeting a pedaling frequency of 85 rpm with 20 W resistance, which was also automatically adjusted during each 20 s recovery period. Subjects were given a countdown and remained seated while sprinting “all-out” for 10 s with verbal indication of time during each sprint for pacing prevention. RSE consisted of repetition of short “all-out” efforts (<10 s) with incomplete recoveries (<60 s) (Girard et al., 2012). The present RSE (10 s:20 s) likely induces a high anaerobic contribution as shown by previous research (Girard et al., 2011; Bishop, 2012). It was reported that RSE induces different acute hemodynamic responses when performed to exhaustion either in systemic hypoxia or with BFR (Willis et al., 2017, 2018) with greater changes in total hemoglobin present during BFR conditions (Willis et al., 2019). Both from a mechanistic and a practical point of view, it is of interest to investigate if IPC and HPC would also induce greater changes in the hemodynamic response of deoxygenation and total hemoglobin during RSE.

### Physiological Variables Measurements

#### Oxygen Uptake Consumption (V̇O_2_, ml⋅min^–1^)

The breath-by-breath gas exchange analysis system (Oxycon Pro Jaeger, Viasys Healthcare GmbH, Höechberg, Germany) was calibrated first with gas calibration (5.03% CO_2_, 15.06% O_2_, PanGas AG, Dagmersellen, Switzerland). Then, air volume was calibrated using a 3-l calibration syringe (M9474, Medikro Oy, Finland). During RSE, the peak ventilation (V̇O_2__peak_) was measured for each of the eight sprint bouts, with the highest 30-s average recorded.

#### Oxygen Saturation (SpO_2_,%)

The SpO_2_ of the left earlobe was recorded at 5-s intervals with an oximeter (8000Q2 Sensor, Nonin Medical Inc., Amsterdam, Netherlands). The data were then downloaded on *nVision* software (Nonin Medical, Inc., Minneapolis, MN, United States). The measurement was analyzed at the end of each preconditioning cycle (during the last 30 s of each stage), during RSE, the minimum value of each sprint was analyzed. Mean oxygen saturation during the entire RSE also was calculated for each condition.

#### Heart Rate (HR, bpm)

Heart Rate was monitored at 5 s intervals (Polar RS400, Kempele, Finland) and subsequently analyzed using the Polar Pro Trainer 5 software (Polar Electro GmbH, Büttelborn, Germany). During the preconditioning protocol, mean values of the final 30 s of each of the four cycles were calculated. During RSE, peak HR of each sprint and mean HR during the last 30 s of RSE were used for the analysis.

#### Blood Lactate Concentration ([La])

The right earlobe was cleaned and pricked to obtain a small blood droplet for analysis as measured with a handheld device (Lactate Scout, SensLab, GmbH, Leipzig, Germany) during the last minute of each preconditioning session. The same procedure was repeated just before starting and immediately after the RSE.

#### Repeated-Sprint Exercise

Maximal and mean power output (W) were determined for each sprint. Mean values over eight sprints were then calculated for each condition. Sprint decrement score was calculated using the formula: (Sprint decrement score (%) = [1 – ((S_1_ + S_2_ + S_3_ + . + S_8_)/(S_best_ × 8))] × 100), where S1 corresponds to sprint 1 mean power, etc, and S_best_ is the best sprint time (usually the first repetition).

#### Near-Infrared Spectroscopy (NIRS)

**FIGURE 1:**
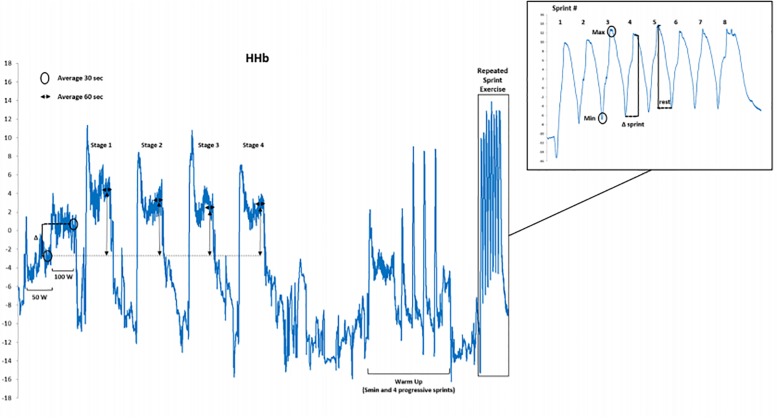
Illustration of the methodology and normalization procedure of NIRS with one subject’s raw signal of deoxygenation (HHb).

During RSE, muscle oxygenation trends were measured continuously using the NIRS technique to calculate tissue concentrations in oxyhemoglobin ([O_2_Hb], μm), deoxyhemoglobin ([HHb], μm), total hemoglobin ([tHb], μm), and tissue saturation index (TSI,%). A PortaMon NIRS device (PortaMon, Artinis, Zetten, Netherlands) was placed on the vastus lateralis (VL) muscle at one third of the distance from the patella to the greater trochanter of the femur. The unit was carefully covered in a transparent plastic wrap to avoid humidity influencing the signal and maintain a waterproof barrier for the device’s function. Using a permanent pen, probe placement was precisely marked following each test visit to ensure the same placement during each visit. Measurements included a standard differential pathlength factor of 4.0 for the VL as there is a lack of any clear standard value during cycling (Faiss et al., 2013), and unknown significance of any error in its variation (Barstow, 2019). The continuous wave NIRS device provides changes in concentration with respect to an initial baseline value (see details below) (Barstow, 2019). The NIRS has demonstrated very high reliability regarding muscle oxygen consumption during low- to moderate-intensity exercise (Lucero et al., 2018). Furthermore, the day-to-day variation in these parameters is about 8.0–9.4% (Kishi et al., 2003; Kolb et al., 2004). All signals were recorded and exported at 10 Hz for analysis (Oxysoft 3.0.53, Artinis, Netherlands). For analysis, a 4th-order low-pass zero-phase Butterworth filter (cutoff frequency 0.2 Hz) was applied to reduce artifacts and smooth perturbations in the signal from pedal strokes (Rodriguez et al., 2018). Regarding the preconditioning analysis, data were expressed as a change implying that each stage (preconditioning cycle) was normalized to a dynamic baseline for interpretation of a stable signal [(average of last 60 s of each stage) - (average of last 30 s of a warm-up stage at 50 W)]; except for absolute TSI values that was expressed as the average of the last 60 s of each stage. For RSE analysis, the changes in concentration (from NIRS) during each sprint were expressed in relation to the change in values between cycling at 100 and 50 W [(min – max of each sprint) − (average of last 30 s of warm-up stage 100 W - average last 30 s of warm-up stage 50 W)]. The purpose of this normalization using pre warm-up values, as performed before the preconditioning, was to allow consistency for between- and within-subjects comparisons of the change in concentration values, since NIRS data represents an arbitrary scale. See Figure 1 outlining the methodology and normalization procedure of NIRS with one subject’s raw signal of [HHb].

### Statistical Analysis

All results are presented as mean ± standard deviation (SD). Following inspection of residual plots, there were no obvious deviation from homoscedasticity or normality. For the preconditioning phase, two-way repeated measures ANOVA [Group (T and UT) × Condition (CON, IPC, HPC, and HIPC)] were applied on dependent variables (HR; SpO_2_; RPE; and [La^–1^]) (Sigma Stat 3.5, Systat Software, San Jose, CA, United States). A linear mixed model similar to a three way repeated-measures ANOVA (Sprint number × Condition × Group) analysis was performed using SPSS (version 22.0, IBM, United States) for repeated sprints variables (Mean power; S_dec_; HR; SpO_2_; RPE; [La^–1^]; V̇O_2__peak_; and NIRS parameters) to account for single missing and random data points (NIRS) due to mechanical error and allow for analysis of the remaining data on the same participants. Based on the ANOVA or linear mixed model analyses, Tables 1, 2 illustrate the “Group main effect” (i.e., difference between the two groups when all four conditions are averaged); the “Condition main effect” (i.e., difference between preconditioning interventions when trained and untrained subjects are averaged) and the interaction between groups x conditions. Fixed effects were identified as sprint number, condition, and group, with participant as the random effect. Differences were considered statistically significant at *p* < 0.05. Pearson correlation coefficients were calculated between the delta in tissue oxygenation index (ΔTSI) after each cycle of the active preconditioning during IPC, HPC, and HIPC conditions and ΔTSI along the repeated sprint exercise following each treatment.

**TABLE 1:** Cardiorespiratory and perceptual data from preconditioning stages in control (CON), ischemic preconditioning (IPC), hypoxic preconditioning (HPC), and hypoxic ischemic preconditioning (HIPC) conditions in both trained (T) and untrained (UT) cyclists (*n* = 8 trained, *n* = 9 untrained).

		**CON**	**IPC**	**HPC**	**HIPC**	**Group main effect**	**Condition main effect**	***P*, interaction**
Mean HR (bpm)	T	113 ± 7	129 ± 10	125 ± 7	145 ± 13	★	^∗^ # & ‡	*P* = 0.939
	UT	124 ± 6	143 ± 10	140 ± 10	156 ± 10	*P* = 0.008		NS
Overall RPE	T	9.8 ± 1.7	12.3 ± 2.1	10.3 ± 1.5	12.7 ± 1.6		^∗^ # †§	*P* = 0.409
	UT	10.4 ± 1.9	12.5 ± 1.4	11.6 ± 2.0	14.3 ± 1.4			NS
Legs RPE	T	10.1 ± 1.6	14.0 ± 1.4	10.5 ± 1.0	14.0 ± 1.9		^∗^ # †§	*P* = 0.115
	UT	10.1 ± 1.5	14.6 ± 1.4	10.5 ± 1.6	15.8 ± 1.4			NS
Breathing RPE	T	9.9 ± 1.8	11.4 ± 2.2	10.5 ± 1.7	12.7 ± 1.9		^∗^ & #	*P* = 0.205
	UT	10.1 ± 1.7	11.6 ± 1.6	12.3 ± 2.7	12.4 ± 1.9			NS
SpO_2_ (%)	T	95.4 ± 1.1	96.0 ± 1.1	78.8 ± 3.2	84.8 ± 2.7		& †‡	*P* = 0.176
	UT	95.0 ± 1.2	95.2 ± 1.2	80.3 ± 3.5	83.2 ± 2.4			NS
Blood Lactate (mmol⋅L^–1^)	T	0.8 ± 0.2	1.4 ± 0.3	1.0 ± 0.6	2.3 ± 0.5	★	^∗^ ‡	*P* = 0.992
	UT	1.7 ± 0.6	2.0 ± 0.5	1.9 ± 1.1	3.1 ± 0.8	*P* = 0.002		NS
Mean Δ[O_2_Hb] (μm)	T	2.2 ± 3.9	3.2 ± 9.3	−2.3 ± 6.3	−3.3 ± 6.3		& #!	*P* = 0.059
	UT	3.1 ± 3.2	0.3 ± 7.0	−1.9 ± 4.2	−2.8 ± 7.5			NS
Mean Δ[HHb] (μm)	T	1.5 ± 2.2	4.4 ± 2.7	8.2 ± 2.3	7.1 ± 3.0		^∗^ & # †!	*P* = 0.252
	UT	2.9 ± 3.9	5.4 ± 3.3	8.6 ± 3.4	9.0 ± 4.2			NS
Mean Δ[tHb] (μm)	T	3.8 ± 3.3	7.6 ± 11.2	5.9 ± 4.5	3.8 ± 6.6			*P* = 0.051
	UT	6.0 ± 4.8	5.6 ± 7.4	6.7 ± 5.1	6.3 ± 8.2			NS
Mean absolute TSI_max_ (%)	T	64.1 ± 7.1	63.1 ± 6.7^a^	58.7 ± 7.5^a,b^	58.8 ± 6.9^a,b^		NA	*P* = 0.001
	UT	60.6 ± 4.5	58.7 ± 3.9^a^	57.2 ± 3.7^a^	57.7 ± 5.1^a^			*F* = 5.438

*Mean ± SD. HR, heart rate; RPE, rating of perceived exertion; SpO_2_, pulse oxygen saturation; HHb, deoxyhemoglobin; tHb, total hemoglobin; TSI_*max*_, absolute maximal tissue saturation index. Mean values over the four preconditioning stages were calculated for Δ[O_2_Hb], Δ[HHb], Δ[tHb], and absolute TSI_*max*_. ★(*P* < 0.05); significant main effect of group, difference between T and UT for all conditions. ^∗^ (*P* < 0.05); significant main effect of condition, difference between CON and IPC. & (*P* < 0.05); significant main effect of condition, difference between CON and HPC. # (*P* < 0.05); significant main effect of condition, difference between CON and HIPC. †(*P* < 0.05); significant main effect of condition, difference between IPC and HPC. ! (*P* < 0.05); significant main effect of condition, difference between IPC and HIPC. §(*P* < 0.05); significant main effect of condition, difference between HPC and HIPC. ‡ (*P* < 0.05); significant main effect of conditions, difference between all conditions (CON, IPC, HPC) and HIPC. ^*a*^ (*P* < 0.05); significantly different than CON. ^*b*^ (*P* < 0.05); significantly different than IPC. NS: Not significant.*

**TABLE 2:** Cardiorespiratory and perceptual data from repeated sprint exercise (RSE) in control (CON), ischemic preconditioning (IPC), hypoxic preconditioning (HPC), and hypoxic ischemic preconditioning (HIPC) conditions in both trained (T) and untrained (UT) cyclists (*n* = 8 trained, *n* = 9 untrained).

	**CON**		**IPC**	**HPC**	**HIPC**	**Group main effect**	**Condition main effect**	***P*, interaction**
Mean Power (W)	T	787 ± 62	782 ± 52	767 ± 54	786 ± 68	★		*P* = 0.184
	UT	677 ± 111	706 ± 86	695 ± 96	691 ± 92	*P* = 0.034		NS
Sprint decrement score (%)	T	16.2 ± 1.6	17.2 ± 2.9	17.8 ± 5.2	17.0 ± 3.1	(★)		*P* = 0.276
	UT	23.4 ± 9.2	19.3 ± 6.1	19.9 ± 9.9	21.2 ± 5.1	*P* = 0.109		NS
RPE Overall	T	17.0 ± 2.9	17.3 ± 1.5	16.9 ± 2.3	17.0 ± 1.8			*P* = 0.964
	UT	17.7 ± 1.7	18.2 ± 1.9	17.8 ± 2.1	17.9 ± 1.8			NS
Max HR (bpm)	T	179 ± 10	179 ± 11	179 ± 11	180 ± 10			*P* = 0.471
	UT	184 ± 7	182 ± 6	183 ± 6	181 ± 6			NS
V̇O_2peak_ (ml⋅min^–1^)	T	3974 ± 377	3933 ± 263	3937 ± 415	3818 ± 429			*P* = 0.909
	UT	3584 ± 667	3624 ± 545	3554 ± 492	3522 ± 581			NS
SpO_2_ (%)	T	97.9 ± 1.4	93.8 ± 5.2	91.2 ± 8.1	95.5 ± 4.2	^∧^		*P* = 0.011
	UT	93.1 ± 7.0	93.8 ± 6.1	98.2 ± 3.8^a^	94.1 ± 6.5	*P* = 0.019		*F* = 4.17
Blood Lactate (mmol⋅L^–1^)	T	13.9 ± 2.8	14.5 ± 2.2	13.3 ± 2.7	13.6 ± 2.4	(★)		*P* = 0.827
	UT	12.1 ± 2.5	11.5 ± 3.1	11.5 ± 3.1	10.6 ± 3.2	*P* = 0.071		NS
Δ[HHb] (μm)	T	2.9 ± 11.8	7.0 ± 7.8^b^	3.4 ± 7.1^c^	5.9 ± 6.0^b,d^		NA	*P* = 0.001
	UT	6.6 ± 4.6	4.2 ± 3.8	2.9 ± 1.7^b^	2.3 ± 2.2^b^			*F* = 13.52
ΔTSI (%)	T	3.4 ± 10.5	3.5 ± 6.1	−0.2 ± 7.9^b,c^	0.4 ± 7.1^b,c^		NA	*P* = 0.004
	UT	6.6 ± 4.3	3.5 ± 2.2^b^	4.4 ± 4.1^b^	2.3 ± 4.8^b^			*F* = 4.46
Δ[tHb] (μm)	T	−1.5 ± 5.7	−1.8 ± 6.8	−6.0 ± 5.3^b,c^	−0.2 ± 4.4^d^		NA	*P* = 0.001
	UT	−1.0 ± 6.1	−3.4 ± 3.4^b^	−5.4 ± 4.5^b^	−4.1 ± 5.1^b^			*F* = 5.64
Absolute TSI_max_ (%)	T	67.6 ± 6.2	65.5 ± 7.0	64.5 ± 7.0	65.0 ± 6.6			*P* = 0.111
	UT	64.1 ± 4.5	65.1 ± 4.3	64.6 ± 4.4	64.1 ± 4.3			NS

*Mean ± SD. RMS, root mean square; RPE, rating of perceived exertion; HR, heart rate; V̇O_2peak_, peak oxygen uptake; SpO_2_, pulse oxygen saturation; HHb, deoxyhemoglobin; TSI, tissue saturation index; tHb, total hemoglobin; TSI_*max*_, absolute maximal tissue saturation index. ★ (*P* < 0.05), (★) Trend; significant main effect of group, difference between T and UT for all conditions. ^ (*P* < 0.05); significant main effect of group, difference between T and UT for HPC only. ^∗^ (*P* < 0.05); significant main effect of condition, difference between CON and IPC. & (*P* < 0.05); significant main effect of condition, difference between CON and HPC. # (*P* < 0.05); significant main effect of condition, difference between CON and HIPC. †(*P* < 0.05); significant main effect of condition, difference between IPC and HPC. ‡ (*P* < 0.05); significant main effect of condition, difference between IPC and HIPC. §(*P* < 0.05); significant main effect of condition, difference between HPC and HIPC. ^*a*^ (*P* < 0.05); significantly different than T. ^*b*^ (*P* < 0.05); significantly different than CON. ^*c*^ (*P* < 0.05); significantly different than IPC. ^*d*^ (*P* < 0.05); significantly different than HPC. NS: Not significant.*

## Results

### Responses During Active Preconditioning

See Table 1 for physiological and oxygenation responses of the preconditioning stages. In both groups, HR increased similarly during the preconditioning phase in IPC and HPC compared to CON (*p* < 0.001), which was further increased during HIPC (*p* < 0.001) compared to all other conditions. Level of discomfort in the legs and overall RPE were greater during ischemic phases (IPC and HIPC) compared to CON and HPC.

Additionally, regarding oxygenation responses, greater Δ[HHb] were demonstrated with IPC when compared to CON (*p* < 0.001). Moreover, hypoxic conditions (HPC and HIPC) elicited greater Δ[HHb] compared to IPC (*p* < 0.001), while Δ[tHb] remained similar between conditions (*p* = 0.097). There was an interaction for absolute mean TSI values (*p* < 0.001, *F* = 5.438) with trained group having lower absolute TSI values in all conditions, when compared to CON. Compared to the control condition, lower TSI values were recorded in both groups (UT and T) for IPC, HPC, and HIPC conditions. However, TSI was lower in the two hypoxic conditions (HPC and HIPC) compared to the IPC condition only in the T group.

### Physiological Responses During RSE

Physiological data from RSE are illustrated in Table 2. All four conditions induced similar RSE power output performance. Mean power output during the first sprint was significantly higher than all other sprints in all conditions and in both T and UT (*p* < 0.001). T demonstrated higher power output (+10–16%) than UT in all conditions during RSE (787 ± 62 vs. 677 ± 111 W in CON; 782 ± 52 vs. 706 ± 86 W in IPC; 767 ± 54 vs. 695 ± 96 W in HPC; and 786 ± 68 vs. 691 ± 92 W in HIPC, for T and UT, respectively, *p* < 0.001). All conditions induced similar S_dec_ during RSE in both T and UT.

### Oxygenation Responses During RSE

Deoxyhemoglobin was significantly greater in IPC compared to HPC condition (*p* < 0.001). A group × condition interaction (*F* = 4.460, *p* = 0.004) showed a decreased ΔTSI in IPC, HPC and HIPC when compared to CON (*p* < 0.001 to 0.049) in UT, and a greater ΔTSI in CON and IPC than hypoxic conditions (HPC and HIPC) (*p* < 0.001 to 0.002) in T (Table 2). T showed greater Δ[tHb] in IPC than CON (*p* < 0.001) as well as in HPC compared to all other conditions (*p* < 0.001). There were no main effect differences between sprints regarding the changes in oxygenation (Δ[HHb], *p* = 0.604; Δ[tHb], *p* = 0.957, absolute maximum TSI, *p* = 0.222) as well as no interactions between sprint and either group or condition.

### Correlation Between Stages and RSE Responses

**FIGURE 2:**
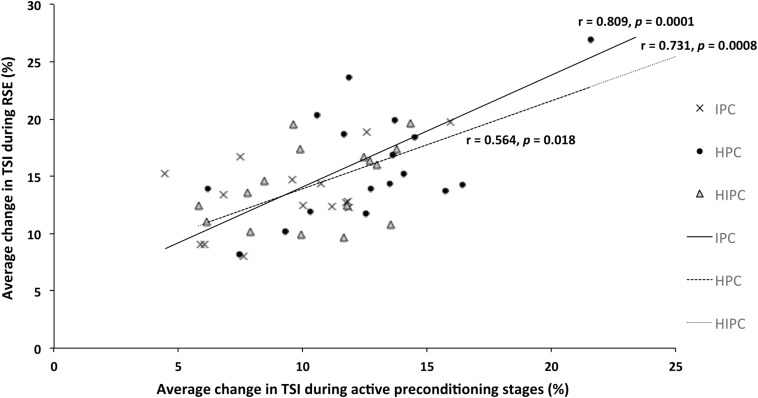
Representation of the relationship between average change (min–max) in tissue saturation index (TSI) amplitude post active phase during four active preconditioning stages and average changes (min–max) of TSI during repeated cycling sprint exercise following each treatment either with partial blood flow restriction (IPC); hypoxia exposure (HPC) or combined hypoxia with partial blood flow restriction (HIPC).

There was a significant correlation between the average change in TSI during active preconditioning stages and average change in TSI during RSE for IPC (*r* = 0.809, *p* < 0.001), HPC (*r* = 0.564, *p* = 0.018), and HIPC (*r* = 0.731, *p* < 0.001), as shown in Figure 2.

## Discussion

The present study evaluated the effectiveness of separate and combined partial blood flow restriction and systemic hypoxic exposure implemented during active preconditioning on repeated-sprint exercise performance and oxygenation responses. None of these active preconditioning techniques (IPC, HPC, or HIPC) led to improved RSE performance either in trained or untrained cyclists, compared to CON.

The first hypothesis was that active preconditioning with systemic hypoxia (HPC) or with BFR (IPC) would lead to larger RSE performance improvement due to larger oxygenation responses (increased oxygen utilization, increased blood volume) than CON or with the combination (HIPC) conditions.

Additionally, it was hypothesized that trained participants would have larger positive oxygenation responses with IPC and even more with HPC, when compared with their untrained counterparts. This hypothesis is partly validated since larger hemodynamic responses were observed in T with IPC and HPC, although it did not lead to improved RSE performance: During repeated sprint cycling exercise, Δ[HHb] (oxygen extraction) was greater in IPC compared to both CON and HPC in T. Further, Δ[tHb] was increased (blood volume) in HPC compared to CON, IPC, and HIPC in T, but increased only in HPC compared to CON in UT.

Finally, the relationship between reoxygenation (change in TSI) responses during active preconditioning (IPC, HPC, and HIPC) phases and during repeated sprint exercise is of interest since an acute response to various preconditioning phases may help to predict the hyperemic (i.e., peripheral vascular function) responsiveness during exercise.

### Preconditioning Phases

The active preconditioning phases resulted in the expected HR and SpO_2_ responses: both IPC and HIPC increased HR compared to CON [i.e., +14% (20 bpm) and +25% (30 bpm)] in both T and UT. These responses may be due to the hemodynamic reaction to blood flow restriction (i.e., drop of stroke volume and cardiac output due to venous return decrease) (Manini and Clark, 2009; Clevidence et al., 2012). To our knowledge, the present preconditioning protocols were novel for many reasons despite the fact that the present study used the most common recommendations for the post-conditioning delay: first, the subjects were active (i.e., cycling at moderate intensity) while previous IPC studies have used passive (i.e., at rest) IPC; secondly, not only did this study assess the separate effects of BFR or systemic hypoxia but also the combined effects of these preconditioning modalities. Hyperemic responses (Δ[tHb]) were observed with active IPC, HPC, and HIPC during the preconditioning phase, which warrants further research on both HPC and IPC. Specifically, more research is needed to establish if these methods could be ergogenic in modifying some parameters, as the number and duration of the preconditioning phases, along with the optimal duration and exercise/rest modalities between preconditioning and RSE.

The occlusion level used for IPC and HIPC in the present study was 60% (124 ± 10 vs. 113 ± 7 mm Hg) of individual maximal occlusion (206 ± 17 vs. 188 ± 11 mm Hg) for T and UT, respectively in order to perform active preconditioning phases. Therefore, the ischemic stimulus in the present study differed from the >220 mm Hg occlusion pressure used in previous studies (Groot et al., 2009; Patterson et al., 2014). For instance, BFR induced an elevation in [La] in IPC compared to CON (*p* = 0.02) and in HIPC when compared to all other conditions (CON, IPC, and HPC, *p* < 0.001). Moreover, IPC and HIPC induced a decrease in TSI_max_ (muscle oxygenation) compared to CON in both T and UT. The incomplete occlusion level (60% of total occlusion pressure) used in the present study is lower than those reported in previous IPC studies (complete occlusion). Therefore, one cannot rule out that this lower occlusion pressure affected muscle oxygenation responses. However, the main aim of the present study was to compare IPC and HPC, while a submaximal occlusion level was selected based on preliminary trials as mentioned earlier to elicit similar muscle deoxgenation between IPC and HPC conditions. However, IPC and HIPC did not induce an increase in blood volume (Δ[tHb]), as expected during total occlusion. Thus, active preconditioning with BFR does not induce the same stimulus as total blood flow occlusion of previous literature and therefore may not induce the same effects on repeated sprint cycling performance. Moreover, it is a common practice to have the sham condition with the cuff inflated to a minimal level (i.e., 30 mm Hg in the present study). However, it remains unclear if it induces or not its own effect during the active preconditioning phase in CON.

This study was the first to test the combination of both ischemic and hypoxic exposure during preconditioning (HIPC) with direct (same participants) comparison of IPC and HPC. This innovative preconditioning technique was more physiologically stressful during the preconditioning stages; i.e., HR and RPE were higher than in all other conditions. Of interest, there was a decrease in TSI as the condition severity increased with both systemic and local hypoxic conditions (i.e., from CON to IPC, HPC, and HIPC) (see Table 1). One may question if these active preconditioning methods induced changes in ischemia-reperfusion or deoxygenation-reoxygenation needed for inducing any effect during RSE. The present data during the preconditioning stages support that active preconditioning induced muscle deoxygenation. There was a decrease in [O_2_Hb] in hypoxic conditions (HPC and HIPC) and absolute TSI in all conditions (IPC, HPC, and HIPC) when compared to CON, along with the important increase in HHb in all conditions compared to CON as well as IPC.

### Repeated Sprint Ability

In the present study, none of the active preconditioning conditions altered repeated sprint cycling performance (mean power output and sprint decrement score). The current results are in agreement with several studies that also failed to demonstrate any beneficial effect of IPC conducted at rest on repeated sprint performance, with six, 6 s sprints and a 30 s Wingate sprint (Lalonde and Curnier, 2015) or five, 6 s cycling sprints (Gibson et al., 2015). Recently, IPC also demonstrated no positive effect on either 16 × 30 m repeated multidirectional sprint exercise performance (Zinner et al., 2017) or 10 and 20 m sprint performance in trained athletes (Thompson et al., 2018). Contrarily, IPC improved peak and mean power of first three sprints during 12 × 6 s repeated sprints (Patterson et al., 2014).

A unique aspect of this study was that preconditioning was conducted actively while exercising on a cycle ergometer. Though these results suggest no benefit of an active preconditioning on RSE performance, previous studies suggest a possible threshold for the amount of tissue under ischemia needed for performance improvement (Kraus et al., 2015). In this way, IPC efficacy would be determined by the volume of tissue exposed to the ischemic stimulus (Loukogeorgakis et al., 2005). Thus, one may speculate that the stimulus was not strong enough or the amount of tissue under ischemia (i.e., muscle mass occluded) was not sufficient in the present study to enhance repeated sprint cycling performance.

It was suggested in a recent review, that a representative time (∼45 min) between IPC and exercise should be adopted in future studies (Salvador et al., 2016). In the present study, the time elapsed between the last preconditioning cycle and the beginning of exercise performance (i.e., 40 min) was therefore in line with Salvador et al. (2016) suggestion. Moreover, the timing between preconditioning and exercise also might influence preconditioning efficacy for short duration exercise (i.e., repeated sprints) (Patterson et al., 2014). Nevertheless, the ideal time between preconditioning and exercise performance remains undetermined.

Since there is greater vascular resistance with 60% of BFR in IPC and HIPC than in CON, there is increased stress placed on the vascular system regarding blood flow regulation and an already decreased arterial oxygen content in the tissue (Willis et al., 2018). Therefore, the compensatory rise in blood flow to muscles due to an additional stimulus of systemic hypoxia during preconditioning (Casey and Joyner, 2012) may not have any additive effect on performance. In fact, there was no difference between HPC and HIPC regarding oxygenation responses of repeated sprint exercise (Table 2). This occurred despite an increase in HHb during the preconditioning stages in IPC compared to CON along with decreased absolute TSI. These hemodynamic responses lead to tissue deoxygenation, and in fact greater deoxygenation in HIPC than IPC alone (Table 1).

### Oxygenation Responses During Sprints

The hypothesis was that trained participants would have larger positive oxygenation responses with IPC and even more with HPC, when compared with their untrained counterparts. This hypothesis was partly validated since larger hemodynamic responses were observed in T with IPC and HPC, although it did not lead to improved RSE performance.

First, Δ[HHb] was greater during RSE in T following IPC. This result is similar to a recent study where increased oxygen extraction (i.e., greater deoxygenated hemoglobin) was observed during a 5 km time-trial in hypoxia following IPC treatment (Wiggins et al., 2019). A larger Δ[HHb] is taken to reflect a greater O_2_ utilization in the exercising muscle (Paradis-Deschênes et al., 2016). It was reported that IPC compared to sham accelerates muscle deoxygenation and influences the improvement in endurance cycling performance (Kido et al., 2015) and maximal contractions (Paradis-Deschênes et al., 2016). Previous research has demonstrated that IPC induced larger vasodilation of about 3% in the brachial artery and therefore greater O_2_ delivery (Enko et al., 2011). In the present study, Δ[HHb] was increased during RSE following IPC in trained group, suggesting a greater oxygen utilization during repeated cycling sprints.

Second, a lower ΔTSI during RSE was reported in hypoxic conditions (HPC and HIPC) compared to CON and IPC in both groups, which was exaggerated in the trained group. Tissue saturation index reflects tissue oxygenation as it corresponds to the dynamic balance between O_2_ supply and utilization (Van Beekvelt et al., 2001; Ferrari et al., 2004). The trained group also had 7% lower arterial blood oxygen saturation (SpO_2_) during RSE than UT following HPC. This observation is in line with greater SpO_2_ desaturation due to exercise-induced arterial hypoxemia, as commonly observed in elite endurance athletes during exercise (Dempsey and Wagner, 1999; Powers et al., 2012). Furthermore, this is related to high cardiac outputs and reduced transit time for oxyhemoglobin loading (Dempsey and Wagner, 1999; Powers et al., 2012). Moreover, the relationships between changes in TSI during preconditioning stages (IPC, HPC, and HIPC) and during RSE suggest possible improvement in peripheral vascular function sensitivity after these preconditioning techniques as recently demonstrated by Da Mota et al. (2019).

Third, all conditions increased Δ[tHb] compared to CON in UT (Table 2), suggesting larger alterations in blood volume (Van Beekvelt et al., 2001) post-preconditioning in this group. HPC induced the greatest Δ[tHb] indicating greater changes in blood volume during RSE following hypoxia exposure in both groups and exaggerated in the trained participants, compared with CON and IPC. In line with the present findings, systemic hypoxia is known to induce a compensatory rise in blood flow to the muscles (Casey and Joyner, 2012). Moreover, it was also shown that repeated sprint training in hypoxia increases Δ[tHb] when compared to normoxia (Faiss et al., 2013). The present results suggest a specific effect of systemic hypoxia exposure on changes in blood volume (i.e., Δ[tHb]) during repeated cycling sprints. Importantly, Δ[tHb] is not an indicator of increased perfusion or blood flow through the muscle vasculature, but merely represents accumulation of blood into the muscle tissue.

Altogether, HPC may have influenced the re-oxygenation and recovery rates during RSE due to greater changes in blood volume. These greater changes in blood volume (i.e., Δ[tHb]) in HPC than other conditions may elicit higher perfusion pressure and improve the diffusion of oxygen delivery to working tissues, thus allowing greater oxygen extraction. This was observed in HPC especially in the trained participants, and in all conditions (IPC, HPC, and HIPC) in UT. Trained participants elicited specific responses in the VL during RSE regarding greater oxygenation utilization and greater changes in blood perfusion depending on the preconditioning treatment.

## Conclusion

In conclusion, local and/or systemic hypoxic stimuli had no ergogenic effect and did not induce any changes in heart rate during cycling RSE. Despite unchanged performance, oxygenation responses were exaggerated in trained compared to untrained cyclists. Despite similar RSE performance across conditions, the muscle oxygenation profile and hemodynamic responses (e.g., oxygen extraction and blood volume) were different between groups and preconditioning methods. Thus, these results suggest that trained cyclists are more sensitive to active ischemic and hypoxic preconditioning stimuli than recreationally active individuals, but without ergogenic effect on repeated sprint cycling performance.

## Data Availability Statement

All datasets generated for this study are included in the article/supplementary material.

## Ethics Statement

The studies involving human participants were reviewed and approved by Ethical Commission for Human Research (CER-VD 138/15). The patients or participants provided their written informed consent to participate in this study.

## Author Contributions

MA, SW, OG, and GM were part of the conception, protocol design, data interpretation, revised critically the manuscript, and gave advises for corrections. MA conducted the experiments and responsible for the data acquisition. FB created the Excel macro in order to analyze NIRS data. MA and SW conducted the analysis and wrote the manuscript. All authors gave the final approval of this version to be published.

## Conflict of Interest

The authors declare that the research was conducted in the absence of any commercial or financial relationships that could be construed as a potential conflict of interest.

## Abbreviations

CON: control condition
HIPC: hypoxic ischemic preconditioning
HPC: hypoxic preconditioning
IPC: ischemic preconditioning
T: trained
UT: untrained.
